# Medication Review by Community Pharmacists for Type 2 Diabetes Patients in Routine Care: Results of the DIATHEM-Study

**DOI:** 10.3389/fphar.2020.01176

**Published:** 2020-08-12

**Authors:** Elisabeth Schindler, Carina Hohmann, Carsten Culmsee

**Affiliations:** ^1^Faculty of Pharmacy, Institute of Pharmacology and Clinical Pharmacy, University of Marburg, Marburg, Germany; ^2^Department of Pharmacy, Klinikum Fulda gAG, Fulda, Germany

**Keywords:** type 2 diabetes, pharmaceutical care, medication review, medication management, drug safety, community pharmacy, interprofessional collaboration, Germany

## Abstract

Most elderly patients with type 2 diabetes take multiple drugs. Earlier studies in other countries suggested that interdisciplinary medication reviews are beneficial for these patients regarding medication safety and therapy optimization. In Germany, medication reviews by community pharmacies are rarely performed, although it is a service stipulated in the “Apothekenbetriebsordnung” (rules governing the operation of pharmacies in Germany) since 2012. Therefore, the aim of the DIATHEM study (type 2 DIAbetes: optimizing THErapy by Medication review in community pharmacies) was to evaluate the impact of medication reviews from German community pharmacies under real-life conditions. Primary outcomes were: identification of drug related problems (DRPs) and to evaluate to what extent they could be solved by the medication review. Secondary outcomes were: evaluation of changes in the number of drugs and the interdisciplinary cooperation between pharmacists and physicians. In a single arm interventional study, 121 patients aged 65 or older with type 2 diabetes, taking at least five drugs for long-term treatment were provided with one medication review between February 2016 and April 2017. Physicians were not pre-informed about the review and neither patients nor physicians nor the 13 participating community pharmacies were reimbursed for their contributions to the study. For 121 patients, 586 DPRs were identified (4.84 DRPs per patient) of which 31.6% were related to the antidiabetics. Due to the medication review, 46.9% of these DRPs could be completely resolved, indicating a statistically significant decline from 4.84 DRPs to 2.57 DRPs per patient (p < 0.001). The average number of drugs was significantly reduced from 9.5 drugs (standard deviation, SD = 2.9) to 9.3 drugs (SD = 2.8) per patient (p < 0.001). The pharmacists received feedback for 76.7% of the intervention proposals sent to the physicians. In total 59.5% of the intervention proposals were accepted, of which 643 (85.3%) were accepted and fully implemented. In conclusion, the study shows that medication reviews performed by community pharmacists under routine care conditions reduced the frequency and number of DRPs, even though the pharmacies had to face obstacles such as lack of cooperation by the prescriber or lack of reimbursement.

## Introduction

Type 2 diabetes (T2DM) is a widespread disease with severe complications. In 2017, approximately 425 million adults worldwide and 58 million Europeans were diagnosed with diabetes and 4 million deaths were caused by this disease. The percentage of T2DM in all diabetes patients is around 90%, but currently increasing in most countries (International DiabetesFederation, 2017). In the US, 30.3 million people (9.4% of the population) suffered from diabetes in 2015 (Centers for Disease Control and Prevention, 2017). In 2017, around 7.5 million adults aged 20-79 in Germany were diagnosed with diabetes, plus an estimated 2.6 million with undiagnosed diabetes (International DiabetesFederation, 2017). T2DM has a prevalence of 7%-8% in German adults, with the highest prevalence for people aged 80 or older (currently around 1 million) (DDG Deutsche Diabetes Gesellschaft, 2018). There are many chronic complications of diabetes such as coronary artery disease and peripheral artery diseases leading to myocardial infarction, stroke, and diabetic foot. Cardiovascular and renal complications are the main causes of death in diabetes patients (International DiabetesFederation, 2017). Mortality rates for people with diabetes (15.6 per 1.000 person-years) are similar to those with a history of stroke or myocardial infarction at the age of 60 years. Without any of these conditions the mortality risk is 6.8 per 1.000 person-years, while any combination of these three conditions further multiplies the risk (The Emerging Risk Factors Collaboration, 2015). Appropriate treatment can avoid these complications and risks. Pharmaceutical care interventions such as a medication review (MR) for patients with T2DM (Wermeille et al., 2004; Machado et al., 2007) and multimorbidity (Koberlein-Neu et al., 2016) may largely contribute to reach that aim. It was shown before that the effect of a MR correlates with the number of drugs in use (Rose et al., 2016).

A MR is “a structured evaluation of a patient’s medicines with the aim of optimizing medicines use and improving health outcomes. This entails detecting DRPs and recommending interventions” (Griese-Mammen et al., 2018). The positive impact of pharmacist-led interventions such as MR on medical treatment and quality of life was repeatedly shown in international studies (Isetts et al., 2003; Isetts et al., 2008; Laaksonen et al., 2010; Pringle et al., 2014; Chau et al., 2016). Also in the field of diabetes, international investigations demonstrated the positive impact of pharmacist-led interventions on diabetes patients by reduction of HbA1c (Wermeille et al., 2004; Machado et al., 2007; Anaya et al., 2008; Wubben and Vivian, 2008), blood pressure (Wermeille et al., 2004), blood glucose (Anaya et al., 2008), lipid profile (Wermeille et al., 2004; Anaya et al., 2008) as well as the impact on cost reduction (Anaya et al., 2008). In Germany, MRs are at present not comprehensively implemented in community pharmacies, even though it is officially defined as a pharmaceutical service according to the “Apothekenbetriebsordnung” (Vierte Verordnung zur Änderung der Apothekenbetriebsordnung, 2012) (the rules governing the operation of pharmacies) since 2012. Apart from some pilot studies or regional projects (Koberlein-Neu et al., 2016; Seidling et al., 2017; Aktas, 2019; ARMIN, 2019), no remuneration is provided for the community pharmacies by the German healthcare system as an incentive for them to perform MRs.

The aim of the DIATHEM study (type 2 DIAbetes: optimizing THErapy by Medication review in community pharmacies) was to examine the effect of MRs in community pharmacies under routine care conditions in Germany. The influence of MRs performed in a real-life setting on elderly multimorbid patients with TD2M on the frequency of DRPs and on the number of drugs as well as the extent of interprofessional collaboration between pharmacist and physicians were analyzed.

## Materials and Methods

This study was designed as a single arm interventional trial (Dickson et al., 2020).

### Primary Outcomes

Primary outcomes were the identification of type and frequency of DRPs. In particular, the primary outcomes were assessed as type and frequency of DRPs that could be totally, partially, or not at all solved by the MR.

### Secondary Outcomes

Secondary outcomes were the identification of type and frequency of antidiabetic drugs and co-medication, evaluation of changes in the number of drugs by the MR and evaluation of the interprofessional cooperation between pharmacists and physicians in the context of the MR, for example willingness to cooperate in general and acceptance or refusal of intervention proposals.

### Sample Size Calculation

The sample for the primary outcome (reduction in the number of DRPs) was calculated assuming a similar reduction of DRPs as in a comparable study, where a reduction of 0.45 DRPs was shown (Koberlein-Neu et al., 2016). Thus, when comparing the number of DRPs per patient before and after the intervention MR, the aim was an average reduction of 0.5 DRP per patient with an estimated standard deviation of differences of 2.2. Considering 80% power and 5% significance level (alpha) in a paired means power analysis, a minimum of 117 participants were needed (Zar, 1984; Machin et al., 1997).

### Setting

For this one arm interventional trial, type 2 diabetes patients matching the inclusion criteria were recruited in 13 community pharmacies in the area of Munich.

Prior to recruiting, employees (including at least one pharmacist per community pharmacy) of all participating community pharmacies were trained in the study procedure by the study coordinator. The study coordinator (consultant pharmacist) is a pharmacist herself and has acquired additional qualifications and professional experience in the field of medication safety and MR.

The trial patient recruitment started in February 2016 until March 2017. Patients were contacted for recruiting purposes either *via* telephone, post or in person while visiting the community pharmacy. Only patients who were known to the pharmacist to suffer from T2DM or patients who had a prescription for an antidiabetic (excluding insulin) were contacted for recruitment. The MR was carried out during or after a patient’s pharmacy visit.

All patients meeting the inclusion criteria were provided with a MR, type 2a or 2b according to the definition of the PCNE (Griese-Mammen et al., 2018). For a MR type 2a, information is evaluated from medication history and patient interviews, for type MR 2b medication history and clinical data were assessed (Griese-Mammen et al., 2018). DRPs can be potential (in future possibly leading to real problems for the patient) or manifest (the problem already has an impact on the patient and his or her therapy) (Pharmaceutical Care Network Europe, 2019a). One MR per patient was performed.

#### Inclusion Criteria

Inclusion criteria were a patient’s age of 65 years or older, diagnosis of T2DM, treatment with at least five different drugs regarding the total medication (prescription-only or over-the-counter drugs) as long-term treatment (drug use for more than 14 days) including at least one antidiabetic drug and that they could speak German. Either a drug that was taken as long-term medication or a drug that was given on-demand was counted as one drug, but a patient with four drugs as long-term treatment and one drug on-demand for example did not meet the inclusion criteria. The limit value five or more drugs was chosen because polypharmacy, in the majority of cases, is defined as the routine use of five or more medications (World Health Organization, 2019).

#### Data Collection

Patients that met the inclusion criteria were invited to participate in the study by the participating community pharmacies. They had to sign a written declaration of consent for the MR. A documentation sheet with the patient’s data and medication was completed by the patient and discussed with a pharmacist during an appointment in the community pharmacy and forwarded to the consultant pharmacist.

If a patient met the inclusion criteria but was being cared for by an outpatient nursing service and, therefore, unable to visit the community pharmacy by themselves, communication took place between the community pharmacy and the outpatient nursing service. In this case, a detailed medication plan was presented to the community pharmacy, which was then forwarded to the consultant pharmacist.

Patient-related data was collected including name, sex, age, main health complaints, allergies, and medication plan, if available. For each drug the following aspects were recorded: drug name (brand), active ingredient, dosage form, strength, dosage regimen, dosage recommendation, indication for drug if known by the patient, prescribing physician (if the drug was prescribed).

Any additionally available treatment-related information as prescribed by the patient was documented, e.g. laboratory values, vital parameters and diagnosis. If a patient had been prescribed an antidiabetic (oral or injectable, except insulin) and in conversation with the pharmacist stated that he or she was suffering from T2DM, we assumed that this was indeed the truth. In most cases, a medication review was sent to the patient’s physician and we presume that the physician would have informed us if the diagnosis was incorrect.

Drugs were coded with the Anatomical Therapeutic Chemical (ATC) Classification System (WHO Collaborating Centre for Drug Statistics and Methodology, 2019).

#### Intervention

After data collection, the consultant pharmacist performed a PCNE type 2a MR for patients who had an appointment at the community pharmacy, respectively type 2b for those patients supported by an outpatient nursing service. The pharmaceutical personnel of the community pharmacy were informed about any detected DRPs and intervention proposals in order to solve the DRPs by the consultant pharmacist.

The DRPs were intended to be solved in cooperation with the responsible physician except where the nature of the DRP could be resolved by direct interaction between pharmacist and patient. Otherwise, the physicians were contacted in writing (via fax or post) by the community pharmacy and provided with a detailed description of the detected DRPs as well as intervention proposals and feedback requests. No clinical investigators were recruited to the study. The physicians in our study were not informed in advance about this pharmaceutical service, in order to avoid prescription bias or bias by being on the alert for DRPs in advance of the intervention. Another reason why the physicians were not specifically asked to participate in advance was to avoid bias in terms of cooperation or refusal to cooperate with the pharmacists.

If the physician responded, the community pharmacy generated an updated medication plan including any accepted intervention proposals or other changes made by the physician. If there was no response, the physician was contacted a second time, after 2 weeks at the latest, and once again asked for feedback. By non-response after the second inquiry, a medication list was generated on the basis of information exclusively given by the patient or outpatient nursing service.

In a concluding appointment with a pharmacist in the community pharmacy, the patient was provided with the updated medication plan and respective medication list, changes in the medication—made by the physician—were explained, interventions were proposed to the patient and the patient’s questions were answered. According to German law, some interventions, such as altering prescription medication, is not allowed to be done by pharmacists. Therefore, interventions that were proposed to the patient by the pharmacist involved only those interventions that are possible in a German community pharmacy, for example interventions concerning the dose timing of a drug or changes regarding over-the-counter drugs, which have not been prescribed or recommended by a physician. If the patient was supported by an outpatient nursing service, the service personnel were provided with the updated medication plan or medication list. The patient or nursing service was asked to present the medication plan or medication list at the following medical appointment.

As a follow-up and for evaluating the status of the DRPs after the MR, patients were asked about the implementation of proposed interventions and any further medication changes by their physician relating to the MR. The follow-up took place at one of the patient’s next visits after the MR in the community pharmacy or by telephone within 4 weeks after the MR. The involved outpatient nursing services were asked regularly about updated medication plans for patients that were provided with a MR. The final follow-up data was collected in April 2017. Depending on the patient, the time between the concluding appointment, when the medication plan or medication list was handed over to the patient, and the follow-up varied considerably and was not evaluated. The last patient was enrolled in the study at the beginning of April 2017. It is a coincidence that the follow-up for this patient also took place in April 2017 and not later.

#### Financial Compensation

Patients did not have to pay for this pharmaceutical service and were not paid for participating in the study. Neither the community pharmacies nor the physicians were reimbursed for participating in the study or performing the MR.

### Detection and Classification of DRPs

Prescription-only drugs or drugs only available *via* pharmacies were considered for analysis. Homeopathic drugs were not taken into consideration.

DRPs were categorized using the PCNE (Pharmaceutical Care Network Europe) classification for Drug-Related Problems V9.0 (Pharmaceutical Care Network Europe, 2019b). Drug-drug interactions were identified using the drug interaction module of the German ABDA database in all medications. Regarding ADRs (adverse drug reactions) and drug interactions, only ADRs and drug interactions that were considered clinically relevant were categorized as a DRP.

### Data Analysis and Statistics

De-identified data was used for evaluation. Sample size calculation was determined with PASS 14 Software (PASS, 2015). Statistical analysis was performed using SPSS Statistics version 24 (IBM Corp, Armonk, NY, USA). Test of normal distribution was performed using the Kolmogorov-Smirnov goodness of fit test. The non-parametric two-sided Wilcoxon signed-rank test was used to examine the differences in frequency of DRPs and number of drugs before and after the MR.

Differences with a P value less than 0.05 were considered significant in all tests. Descriptive analysis was performed to examine:

- Demographic data
- Drugs used: antidiabetic drugs and co-medication (examination of the active ingredients, no distinction between originator and generics)
- Type and frequency of identified DRPs
- Drug interactions
- Changes in the number of drugs by the MR
- Type and frequency of identified DRPs that were totally, partially or not at all solved by the MR
- Measures that were taken to solve the DRPs
- Interprofessional collaboration: Proportion of physicians cooperating with the pharmacists in the context of the MR and extent of cooperation.

### Ethics Approval

Before the beginning of the study, the responsible ethics committee was provided with a detailed project description. As the research project was classified as quality assurance, the ethical committee of the Bavarian State Chamber of Medicine (Bayerische Landesärztekammer) stated, that no ethic vote was required for this study.

Only patients that had signed a written declaration of consent for data-retention in the associated community pharmacies were recruited for the study. To participate in the study, the patients had to sign an additional written declaration of consent for the MR. This declaration of consent included the release from the confidentiality obligations for the community pharmacy and the patient’s treating physician(s).

## Results

### Patient Population

Overall, 121 patients were included in the DIATHEM study. More information on baseline characteristics are presented in **Table 1**.

**Table 1:** Demographic and baseline characteristics of patients.

Type 2 Diabetes Patients	
*N*	121
Gender (male/female; percentage)	58 (47.9)/63 (52.1)
Age at examination (years mean ± SD)	76.7 ± 7.6
Age 65-79 (years)	66.9
Age ≥ 80 (years)	33.1
Patients supported by an outpatient nursing service (percentage)	24.8
Involved community pharmacies (number)	13

SD, standard deviation.

### Analysis of Drugs Used

Taken together the 121 patients received 1.149 drugs, of which 221 drugs were diabetic-related (oral or injectable antidiabetics including insulins).

#### Antidiabetics

Of the 221 antidiabetic drugs, biguanides with 43.0% (95 times; only Metformin) were most common, followed by Dipeptidyl-peptidase-4 inhibitors with 21.7% (48 times: Sitagliptin or Saxagliptin), insulins with 15.8% (33 times: most frequent: insulin glargine and insulin detemir) and sulfonylureas with 10.0% (22 times; Glimepirid, Glibenclamid, Gliquidon). Only one insulin or insulin analogue and no other antidiabetic drug was received by two patients (1.7%).

Overall, almost half of all patients (47.1%) took only one antidiabetic drug, while 28.1% of the patients took two antidiabetics, 20.7% three antidiabetics, 3.3% four antidiabetics, and 0.8% five antidiabetics.

#### Co-Medication

In total, 928 drugs (80.8% of all drugs) were assigned to co-medication. The five most frequent drug classes classified by ATC-level 2 (WHO Collaborating Centre for Drug Statistics and Methodology, 2019) were agents acting on the renin-angiotensin system (C09), diuretics (C03), lipid modifying agents (C10), antithrombotic agents (B01), and beta blocking agents (C07), see **Table 2**.

**Table 2:** Shows the number and frequency of the ten most frequent drug classes (classified by ATC-Code level 2 (WHO Collaborating Centre for Drug Statistics and Methodology, 2019)) and the number and frequency of the most frequent drugs from each drug class out of all drugs (1.149).

Drug class(ATC-Code level 2)	Number of drugs in this drug class	Frequency of drugs in this drug class [%]	Drug name	Number of this drug	Frequency of this drug [%]
Antidiabetics (A10)	221	19.2	Metformin	95	8.3
			Sitagliptin	46	4.0
			Insulin	35	2.9
			Glimepiride	19	1.7
			Other	26	2.4
Agents acting on the renin-angiotensin system (C09)	100	8.7	Ramipril	51	4.4
			Valsartan	14	1.2
			Candesartan	13	1.1
			Other	22	1.9
Diuretics (C03)	95	8.3	Hydrochlorothiazide	50	4.4
			Torasemide	30	2.6
			Spironolactone	9	0.8
			Other	6	0.5
Lipid modifying agents (C10)	86	7.5	Simvastatin	53	4.6
			Atorvastatin	22	1.9
			Pravastatin	4	0.3
			Other	7	0.6
Antithrombotic agents (B01)	85	7.4	Acetylsalicyclic acid	58	5.0
			Phenprocoumon	13	1.1
			Rivaroxaban	6	0.5
			Other	8	0.7
Beta blocking agents (C07)	63	5.5	Bisoprolol	43	3.7
			Metoprolol	17	1.5
			Nebivolol	2	0.2
			Other	1	0.1
Drugs for acid related disorders (A02)	51	4.4	Pantoprazole	41	3.6
			Omeprazole	8	0.7
			Hydrotalcite	1	0.1
			Other	1	0.1
Calcium channel blockers (C08)	45	3.9	Amlodipine	37	3.2
			Lercanidipine	4	0.3
			Nitrendipine	3	0.3
			Other	1	0.1
Thyroid therapy (H03)	39	3.4	Levothyroxine Sodium	38	3.3
			Carbimazole	1	0.1
Analgesics (N02)	38	3.3	Metamizole Sodium	12	1.0
			Tilidine	10	0.9
			Acetylsalicyclic acid	4	0.3
			Other	12	1.0
Other drug classes	326	28.4			

### Type and Frequency of Identified DRPs

**Figure 1:**
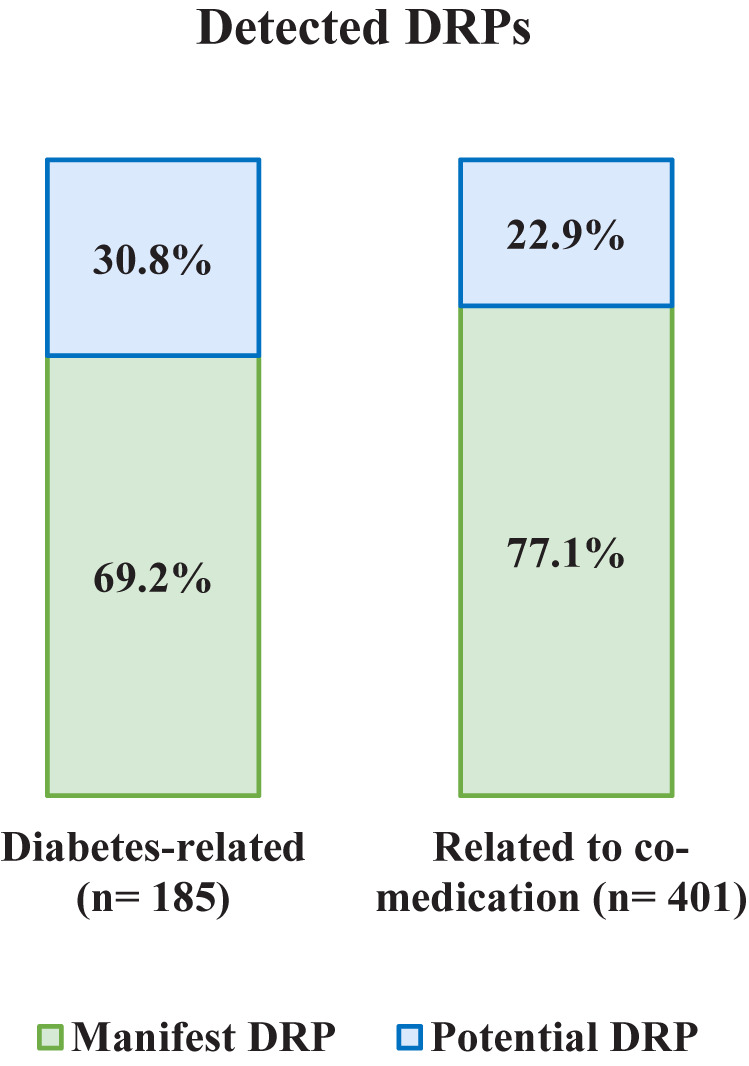
Frequency and type of detected DRPs: manifest and potential, diabetes-related, and related to co-medication.

A total of 586 DRPs were discovered in 121 patients, corresponding to 4.84 DRPs per patient. No DRP was identified in only three patients (2.5%). Of all 586 DRPs, 401 DRPs involved the co-medication (68.4%) and 185 DRPs were diabetes-related. A DRP was assigned to the category diabetes-related if an antidiabetic drug (oral and injectable antidiabetics including insulin) was involved, for example a drug-drug interaction between an antidiabetic drug and an antihypertensive drug was categorized as diabetes-related. Categorized as manifest were 74.6% of all DRPs, the others were potential DRPs (**Figure 1**).

Classified by the PCNE classification for DRPs V9.0, the majority of DRPs were assigned to the problem domain *P1—Treatment effectiveness* (53.6%) and related mainly to the subdomain *P1.2 Effect of drug treatment not optimal* (45.9%). Second most prevalent problem domain was *P2—Treatment safety* (42.0%), related to the subdomain *P2.1—Adverse drug event (possibly) occurring* (42.0%) (**Table 3**). Most often, a DRP was assigned to the sub-section *P1.2 Effect of drug treatment not optimal* (45.9%). Although information on blood glucose level and HbA1c value were in most cases not available, an assignment to this category was possible for diabetes-related DRPs based on information provided by the patient and/or—if available—the medication plan. For example, if a patient was treated with Metformin 2.0 g once a day the code *P1.2 Effect of drug treatment not optimal* was chosen and a change to Metformin 1.0 g twice a day instead of 2.0 g once a day was recommended to the physician.

**Table 3:** Classification of the DRPs—Problem domain.

Classification of the DPRs (n = 586)	Number of problems	Frequency of problems [%]
**P1—Treatment effectiveness**	**314**	**53.6%**
P1.1—No effect of drug treatment	27	4.6%
P1.2—Effect of drug treatment not optimal	269	45.9%
P1.3—Untreated symptoms or indication	18	3.1%
**P2—Treatment safety**	**246**	**42.0%**
P2.1—Adverse drug event (possibly) occurring	246	42.0%
**P3—Other**	**26**	**4.4%**
P3.1—Problem with cost-effectiveness of the treatment	6	1.0%
P3.2—Unnecessary drug-treatment	20	3.4%

For all 586 DPRs, 617 causes have been identified, mostly categorized into C1—Drug selection (45.2%) and C3—Dose selection (49.3%). **Table 4** shows the detailed distribution of the causes.

**Table 4:** Classification of the DRPs—Causes domain.

Causes of DRPs (n= 617 causes for 586 DRPs)	Number of causes	Frequency of causes [%]
**C1—Drug selection**	**279**	**45.2%**
C1.1—Inappropriate drug according to guidelines/formulary	74	12.0%
C1.2—Inappropriate drug (within guidelines but otherwise contra-indicated)	93	15.1%
C1.4—Inappropriate combination of drugs, or drugs and herbal medications, or drugs and dietary supplements	78	12.6%
C1.5—Inappropriate duplication of therapeutic group or active ingredient	8	1.3%
C1.6—No or incomplete drug treatment in spite of existing indication	22	3.6%
C1.7—Too many drugs prescribed for indication	4	0.6%
**C3—Dose selection**	**304**	**49.3%**
C3.1—Drug dose too low	22	3.6%
C3.2—Drug dose too high	88	14.3%
C3.3—Dosage regimen not frequent enough	35	5.7%
C3.4—Dosage regimen too frequent	86	13.9%
C3.5—Dose timing instructions wrong, unclear or missing	73	11.8%
**C4—Treatment duration**	**26**	**4.2%**
C4.1—Duration of treatment too short	1	0.2%
C4.2—Duration of treatment too long	25	4.1%
**C7—Patient related**	**2**	**0.3%**
C7.5—Patient takes food that interacts	2	0.3%
**C9—Other**	**6**	**1.0%**
C9.1—No or inappropriate outcome monitoring (incl. TDM)	6	1.0%

In total, 1.267 interventions were made or proposed by the pharmacist, on average 2.2 interventions per DRP, referring to categories I1—At prescriber level (40.9%), I2—At patient level (22.6%) and I3—At drug level (36.5%). No intervention was made or proposed for 32 DRPs. For example, an intervention proposal was made to the prescriber (code I1.3) if a patient seemed to suffer from T2DM and hypertension, but received no angiotensinconverting enzyme inhibitor (ACE inhibitor) or angiotensin II receptor blockers (ARB). The suggestion was to prescribe an ACE-inhibitor or ARB if there was no contraindication or intolerance as these drugs are recommended by guidelines for patients with T2DM and hypertension due to their nephroprotective potential. **Table 5** shows the detailed distribution of the interventions.

**Table 5:** Classification of the DRPs—Intervention domain.

(Planned) Interventions (n = 1267 interventions for 586 DRPs)	Number of (planned) interventions	Frequency of (planned) interventions [%]
**I0.1—No Intervention**	**32**	**–**
**I1—At prescriber level**	**518**	**40.9%**
I1.1—Prescriber informed only	74	5.8%
I1.3—Intervention proposed to prescriber	384	30.3%
I1.4—Intervention discussed with prescriber	60	4.7%
**I2—At patient level**	**286**	**22.6%**
I2.1—Patient (drug) counselling	236	18.6%
I2.3—Patient referred to prescriber	50	3.9%
**I3—At drug level**	**463**	**36.5%**
I3.1—Drug changed to…	78	6.2%
I3.2—Dosage changed to…	77	6.1%
I3.4—Instructions for use changed to…	180	14.2%
I3.5—Drug paused or stopped	94	7.4%
I3.6—Drug started	34	2.7%

### Drug Interactions

A total of 694 interactions (5.7 per patient) with a minimum of 0 and a maximum of 19 interactions per patient (SD = 4.2), classified by the ABDA database, were identified among the 121 participants. Only 1 of all 694 interactions was within grades 1-3 of the ABDA Database. 11.8% of the interactions were considered clinically relevant and regarded as a DRP in the course of the MR. From the clinically relevant interactions, 80 were drug-drug interactions and two drug-food interactions.

### Reduction of the Detected DRPs

**Figure 2:**
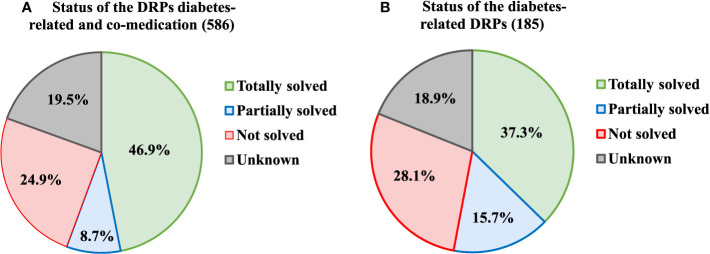
Status of the DRPs after the MR, **(A)** diabetes-related and related to the co-medication, **(B)** diabetes-related only.

In total, 275 out of 586 DRPs (46.9%) could be totally solved. This indicates a statistically significant average decline from 4.84 DRPs to 2.57 DRPs per patient (reduction of 2.27 DRPs per patient, p < 0.001) achieved by the MR. Examining DRPs related to the antidiabetics, 37.3% of the DRPs were completely resolved, which corresponds to an average reduction of 0.96 DRPs per patient. More detailed information on solving the DRPs is provided in **Figure 2**.

### Changes in Number of Drugs

Patients received a total number of 9.5 drugs on average (SD = 2.9). The median number was 9 with a minimum of 5 and a maximum of 19 drugs per patient. Of these drugs, 95.0% (9.0 per patient) were taken as long-term medication, the rest were on-demand medication. Another 19.2% (1,8 drugs per patient) were antidiabetics with a minimum of 1 and a maximum of 5 drugs per patient.

After the MR, patients received a total number of 9.3 drugs on average (SD = 2.8), which indicates a small, but statistically significant lower average number of drugs per patient than before the outset of the MR (p < 0.001). Changes in the number of drugs only affected long-term medication, not on-demand medication. The average number of antidiabetics was reduced by 2.3% (0.04 drugs per patient). The average number of co-medications was reduced by 2.2% (0.17 drugs per patient). It was not evaluated if changes were within the same drug class or between different drug classes.

### Interprofessional Collaboration

#### Physicians’ Feedback on MR

The proportion of physicians cooperating with the pharmacists in the context of the MR and the extent of the cooperation were examined. In this context, the term cooperation was chosen if the physician answered the pharmacist’s question, regardless of whether the intervention proposals were accepted or not. Acceptance of intervention proposals was analyzed separately.

**Figure 3:**
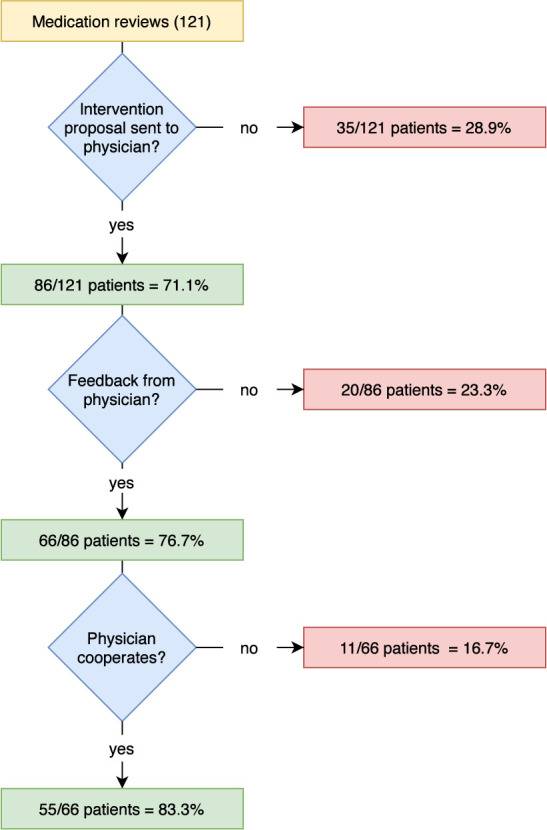
Overview of physicians’ feedback on MR.

For 86 patients (71.1%), intervention proposals for the reduction of detected DRPs were submitted to the responsible physician by the community pharmacy and feedback was requested, see **Figure 3**. In 76.7% of the cases the pharmacy received feedback from the physician, although in almost half of all cases, the pharmacies only received a delayed response after a second feedback request was initiated. No feedback was received for 20 patient cases (23.3%), meaning that the outcome and the status of the DRP after the MR could not be assessed for all DPRs (see **Figure 2**, status of the DRP unknown for 19.5%). In most cases (78.8%), the feedback was given exclusively in writing, sometimes (21.2%) there was also a telephone call between pharmacist and physician. The pharmacy received feedback after an average of 18 days. The overall willingness to cooperate by the physicians involved was 64.0% (55 out of 86 cases where intervention proposals were sent to the physician).

#### Acceptance of Intervention Proposals

More than half of the (59.5%; 754 out of 1267) intervention proposals were accepted, of which 643 (85.3%) were accepted and fully implemented. 220 (17.4%) of the intervention proposals were not accepted, of which 37.3% due to there being no agreement by either the prescriber or the patient and 61.4% for unknown reasons. For 293 (23.1%) of the intervention proposals, the level of acceptance was unknown.

## Discussion

The DIATHEM study aimed to examine the influence of MRs provided by community pharmacies in Germany for elderly type 2 diabetes patients under routine care conditions on type and frequency of DRPs, number of drugs and interprofessional collaboration. Overall, almost half of all DRPs could be totally resolved in the DIATHEM study, indicating a significant reduction of the number of DRPs from 4.84 DRPs to 2.57 DRPs per patient (reduction of 2.27 DRPs per patient).

The benefit of MRs in diabetes patients by community pharmacists in regard to quality of life and drug treatment has already been proven in other countries (Wermeille et al., 2004; Machado et al., 2007; Anaya et al., 2008; Wubben and Vivian, 2008). In the last years, increasing numbers of studies have investigated the positive effects of MRs in Germany, for example in patients with polymedication or with Parkinson’s disease (Henrichsmann and Hempel, 2016; Koberlein-Neu et al., 2016; Müller-Rebstein et al., 2017; Seidling et al., 2017; Aktas, 2019; Bitter et al., 2019). For diabetes patients, a study investigated the impact of pharmaceutical care in adolescents with T1DM (Obarcanin et al., 2015) and another, the GLICEMA study (Schmiedel et al., 2015), showed the significant benefit of a prevention program carried out in German community pharmacies for patients with T2DM. However, to our knowledge, no comparable interventional MR study during the routine care of type 2 diabetes patients in community pharmacies in Germany, has been described to date.

The results from our study demonstrate that elderly type 2 diabetics are multimorbid patients with polymedication leading to a high number of DRPs. This is in concordance with the findings of similar studies, where the number of DRPs was found to be associated with age of the patients and number of drugs (Lenssen et al., 2016). The number of drugs could be marginally reduced in the DIATHEM study, which would have been desirable in terms of patient adherence. However, a relevant reduction in the number of drugs per patient is often impossible to achieve in geriatric patients due to increasing number of different diseases in the elderly. Furthermore, diabetes is often accompanied with severe cardiovascular disease and metabolic syndrome, and patients receive a broad range of drugs for prevention and treatment of this disease. Thus, in the DIATHEM study, the most frequently used drugs belonged to the ATC code group C (cardiovascular system) ahead of the group A (alimentary tract and metabolism, which contains antidiabetics) and N (nervous system). The pattern of the most frequently used drugs is consistent with that of the study by Seidling et al., a post-hoc analysis of MRs provided by community pharmacies in the German ATHINA-project (Seidling et al., 2017). More than 80% of the patients in our study received a drug acting on the renin-angiotensin system, i.e. ACE inhibitors or ARBs. ACE inhibitors respectively ARBs are the preferred drugs for treating hypertension in patients with T2DM because of their nephroprotective potential. Diuretics, lipid modifying agents and beta blockers - drug groups belonging to the group cardiovascular system such as ACE inhibitors and ARBs—were also taken by at least half of the patients. About two-thirds of all patients received one or two antithrombotics, i.e. platelet aggregation inhibitors or anticoagulants. As antithrombotics are high risk drugs, this patient collective from our study is supposed to benefit from a MR as an instrument to improve patient safety. Regarding the antidiabetic medication Metformin was by far the most used drug, received by 78.5% of patients, followed by Sitagliptin (38.0%) and Glimepiride (15.7%). This distribution is consistent with current national guidelines on the treatment of type 2 diabetes. In the German National Care Guideline (Nationale Versorgungs Leitlinie) for therapy of type 2 diabetes, Metformin is still the preferred drug (Bundesärztekammer (BÄK) Kassenärztliche Bundesvereinigung (KBV) Arbeitsgemeinschaft der Wissenschaftlichen Medizinischen Fachgesellschaften (AWMF), 2014). This guideline from 2014 is currently under revision, an update is expected soon (Programm für Nationale VersorgungsLeitlinien, 2019). Changes in the first line recommendations for atherosclerotic and cardiovascular high-risk patients—as already implemented in the 2019 ESC/EASD Guidelines on diabetes, pre-diabetes, and cardiovascular diseases (Cosentino et al., 2019)—are considered possible. The basis for these recommendations is that in recent years there has been new and convincing evidence from cardiovascular safety studies [i.e. LEADER-, SUSTAIN-6-, EMPA-REG OUTCOME-, and CANVAS-study (Zinman et al., 2015; Marso et al., 2016a; Marso et al., 2016b; Neal et al., 2017)] demonstrating mortality benefits for type 2 diabetics with cardiovascular disease or at high cardiovascular risk and treatment with sodium-glucose co-transporter 2 inhibitors or glucagon-like peptide-1 analogues. However, at the time of data collection for the DIATHEM study, these studies had not yet or only recently been published and these findings were not yet integrated in treatment regimens.

DRPs in patients with type 2 diabetes are not only associated with the antidiabetic drugs, but even more with the co-medication. Nearly 70% of all DRPs in our study were related to the co-medication, which corresponds to 3.31 DRPs per patient compared to 1.53 diabetes-related DRPs per patient on average. Only in 2.5% of the patients no DRP could be identified, indicating a high need for MRs in elderly patients with T2DM. Regarding the distribution of DRPs, the most frequently found DPRs concerned drug dosage and drug selection, both in antidiabetics and in co-medication. These findings are in concordance with those of comparable studies, where dosage and drug selection were also among the most frequent detected DRPs (Koberlein-Neu et al., 2016; Bitter et al., 2019). In our study inappropriate dosage affected underdosing, e.g. underdosing of a antidiabetic drug resulting in insufficient blood sugar control, and also overdosing, e.g. no dose adjustment even though it was necessary due to renal failure. Problems in the drug selection process involved combinations of antidiabetics with high risk for hypoglycaemia in patients with a history of hypoglycaemia. Often, no adjustments of drug dosage and drug selection is made during long-term treatment. In such cases, MRs help to discover and resolve those DRPs. Patients should be encouraged to regularly visit their physicians for check-ups and to inform them of side effects. Another frequent DRP in the total medication was an unsuitable or inappropriate dosage regimen. A correct dosage regimen is not only important for the effect of drugs, but also has an influence on possible side effects and on patient adherence. In order to maintain patient adherence at the highest possible level, the number of doses of drugs to be taken daily should be as low as possible, as it is known that adherence decreases with increasing number of doses to be taken (Claxton et al., 2001; Saini et al., 2009). In this respect, the pharmacokinetic knowledge of pharmacists is particularly valuable. Drug-drug interactions were common but not predominant DRPs in the DIATHEM study and in many cases related to risk of bleeding, potassium level and possible QTc-prolongation. Also here, the patient-specific drug selection and regular check-ups play an important role. Pharmacists are trained to recognize and evaluate drug-drug interactions and to develop intervention proposals for treatment optimization and therefore well suited to use these skills in the course of a MR.

This study shows that the intervention MR is a highly effective instrument to reduce DRPs in elderly patients with T2DM. A comparison with studies in similar settings that have investigated the influence of MR provided by community pharmacies in Germany shows that the number of detected and resolved DRPs varies relatively strongly. A recent study from Bitter et al. (mean age 84 years, long-term care residents, MRs provided for 94 patients) found on average 1.6 DRPs per patient, of which one third of the pharmacist’s interventions to resolve the DRPs were successfully implemented (Bitter et al., 2019). The WestGem study (mean age 76.8 years, mean number of drugs 10.5 per patient, MRs provided for 142 patients) showed a mean number of 7.3 DRPs per patient on baseline of which 0.45 DRPs per patient were resolved (Koberlein-Neu et al., 2016). This variety shows that there are currently no standard criteria in Germany for the assessment and classification of DRPs.

Regarding interdisciplinary cooperation, the study shows the immense importance of the interprofessional collaboration between pharmacists and physicians. An enormous amount of DPRs could be resolved by pharmacists and physicians working together in the context of the MRs. The extent of interprofessional collaboration in the DIATHEM study was positive with an overall 64.0% of the physicians willing to cooperate. Furthermore, the overall acceptance of the pharmacist’s intervention proposals by the patients and physicians was 59.5%. The DIATHEM study demonstrates that these results can actually be achieved under routine care conditions. In contrast to our study, existing studies on MR in Germany do not fully reflect the real-life situation in community pharmacies. In most studies, clinical investigators were involved or the physicians were informed in advance about the MR, had agreed to participate in the study and/or were reimbursed for participating. Patients could also be chosen with a bias, i.e. based on the knowledge that the responsible physician was willing to cooperate with the community pharmacy. In our study, patients who met the inclusion criteria could receive a MR and participate in the study, regardless of their physician. Due to the fact that few physicians were aware that the MR is also a service provided by the community pharmacies, it is assumed that involving clinical investigators or having personal contact between pharmacist and physician beforehand would have increased the proportion of physicians agreeing to a collaboration. The fact that there was no reimbursement for any of the interest groups is also believed to have had a negative influence on cooperation. A few physicians even refused to communicate with the community pharmacy during the course of the MR with the explanation that they were not paid for their time investment. These factors, which were challenging in execution of the study, are consistent with findings in international studies evaluating facilitators and barriers within pharmaceutical services in community pharmacies and primary care teams (Roberts et al., 2008; Jorgenson et al., 2014).

Thus, although MR can already be successfully carried out in German community pharmacies within their day-to-day routine, the prerequisites are poor, even though the ABDA has developed a manual for the performance of a MR (ABDA, 2018) and there are some training programs offered for pharmacists such as the ATHINA project (Information on the ATHINA project, 2020) or the Apo-AMTS project (Information on the Apo-AMTS project, 2020). Interprofessional cooperation should be expanded and promoted throughout the whole of the healthcare system, e.g. joint courses during medical and pharmaceutical education as a starting-point. Furthermore, by giving physicians the possibility to prescribe a pharmacist-led MR for patients at risk, working together to solve the prevailing DRPs and appropriate reimbursement for all involved partners. An appropriate reimbursement of costs is also necessary because performing a MR is time consuming. For this service to be widely implemented in community pharmacies, a suitable amount of time must be appointed to staff members in order to perform all duties in context of a MR. Moreover, pharmaceutical education should be extended to enable all pharmacists to conduct MRs without the necessity of additional qualifications. In this study, a consultant pharmacist with broad professional experience and additional education regarding drug safety was responsible for the MRs.

The limitations of this study are mainly attributed to the real-life setting. In daily care conditions in German community pharmacies, the pharmacies do not routinely obtain data on either laboratory values or diagnosis of the patient, including the severity and duration of the diseases. Therefore, we cannot provide detailed information on these particular issues. If patients received an antidiabetic and in conversation with the pharmacist stated that they were suffering from T2DM, we assumed that this was indeed the truth. In most cases, a medication review was sent to a physician and we presume that the physician would have informed us if the listed diagnosis was incorrect. It is also not feasible in routine care conditions to measure HbA1c value and blood glucose for every single patient in addition to the standard care of all patients or to acquire data on patient adherence i.e. pill count. Therefore, information on clinical data that are in general valuable for a study on T2DM patients is missing. The study shows that DRPs were reduced by the MR, but the impact of reduced DRPs on safety and tolerability as well as on morbidity and mortality remains unclear, because it was not evaluated. It was likewise not evaluated if the demonstrated reduction in the number of drugs results in improved clinical outcomes. Another limitation of the study is, when examining the reduction of DRPs and the number of drugs by the MRs, it was only distinguished between diabetes-related drugs and drugs related to the co-medication, but no further differentiation between antidiabetic drugs (injectable versus oral) was made. Further limitations of this study were shown partly by a lack of willingness to cooperate by some of the physicians involved. This led to unsolved DRPs and other DRPs left with an unknown status after the MR. In the study, patients received the service MR at no personal costs. We did not test patient satisfaction with the MR or examine changes in quality of life, and did not evaluate if they would be willing to pay for the service themselves. Furthermore, no adjustments were made for multiplicity of exploratory outcomes.

On a whole, the DIATHEM study demonstrates that structured MRs in elderly patients with type 2 diabetes under routine care conditions in German community pharmacies are successful and shows the high benefit of this service. Enhancement of interprofessional collaboration and pharmaceutical education as well as reasonable remuneration are necessary factors in reducing existing barriers. Data from real-life studies are precious and of great importance in order to improve patient care.

## Data Availability Statement

The datasets generated for this study are available on request to the corresponding author.

## Author Contributions

Study concept and design: ES, CC, and CH. Data collection and analysis: ES. Wrote the paper: ES, CC, and CH.

## Funding

The study was funded by the “Förderinitiative Pharmazeutische Betreuung e.V.” (www.foerderinitiative.de).

## Conflict of Interest

All authors declare that the research was conducted in the absence of any commercial or financial relationships that could be construed as a potential conflict of interest.
